# *Rhadinoscelidia lixa* sp. nov. (Hymenoptera, Chrysididae, Loboscelidiinae) found on an ant nest in Thailand

**DOI:** 10.3897/zookeys.975.54952

**Published:** 2020-10-12

**Authors:** Yu Hisasue, Toshiharu Mita

**Affiliations:** 1 Entomological Laboratory, Graduate School of Bioresource and Bioenvironmental Sciences, Kyushu University, 744, Motooka, Nishi-ku, Fukuoka, Fukuoka, 819-0395, Japan Kyushu University Fukuoka Japan; 2 Entomological Laboratory, Faculty of Agriculture, Kyushu University, 744, Motooka, Nishi-ku, Fukuoka, Fukuoka, 819-0395, Japan Kyushu University Fukuoka Japan

**Keywords:** *Carebara diversa*, chrysidid wasp, myrmecophily, taxonomy, Thailand

## Abstract

*Rhadinoscelidia lixa***sp. nov.** is described from Thailand. It is the fifth species of the genus and second species from Thailand. A biological note on the species with its associated ants is provided.

## Introduction

Loboscelidiinae are rare and morphologically peculiar chrysidid wasps. The subfamily contains two genera, *Loboscelidia* Westwood, 1874 and *Rhadinoscelidia* Kimsey, 1988. To date, *Rhadinoscelidia* is known by four species from Hainan Island (China), Thailand, Laos, West Java (Indonesia), and peninsular Malaysia (Kojima and Ubaidillah 2003; Liu et al. 2011; Kimsey 2018). The genus is similar to *Loboscelidia* in some morphological characters; however, *Rhadinoscelidia* can be distinguished from *Loboscelidia* by the following characters: cervical expansion separated from upper gena with reduced patches of ribbon-like setae, reduced wing venation of the forewing, and reduced flanges on the legs (Kimsey 2018). Although nothing is known about their biology, the genus *Loboscelidia* is considered as an egg parasitoid of stick insects, similar to the Amiseginae, and the bizarre structural modification implies their myrmecophily (Riek 1970; Krombein 1983; Kimsey 2012). During the investigation of chrysidid fauna of Southeast Asia, we had a chance to examine an unidentified female of *Rhadinoscelidia* from Thailand. This wasp was found staying at the nest entrance of the ant species *Carebara diversa* (Jerdon, 1851) (Formicidae, Myrmicinae).

In this paper, we describe it as a new species of *Rhadinoscelidia* and provide a key to known species, and a brief discussion on the life history, of the genus.

## Materials and methods

The material used in this study is deposited in the Entomological Laboratory, Faculty of Agriculture, Kyushu University, Japan. Images were taken with a Canon EOS Kiss X8i camera and edited using Adobe Photoshop CC. Morphological terminology and measurements mainly follows Kimsey (1988, 2018) and Liu et al. (2011). The following abbreviations and indices were used: maximum length of median ocellus diameter (MOD), minimum length of postocellar line (POL), minimum length of ocello-ocular line (OOL), maximum length of lateral ocellus diameter (LOD), lateral ocellar line (LOL, Masner and Huggert 1989) is the shortest distance between the inner margins of median and lateral ocelli, segment of flagellomere (F), metasomal tergite (T), and metasomal sternite (S).

## Taxonomy

### Rhadinoscelidia

Taxon classificationAnimaliaHymenopteraChrysididae

Kimsey, 1988

BD42B9C7-CE2D-593C-88A3-56915382708D

#### Diagnosis.

Antennal scape distinctly longer than head; vertex sharply declivous behind ocelli; cervical expansion of head with posterior shield-like expansion clearly separate from rest of head; forewing venation highly reduced, restricted to basal sixth or less; all tibiae without flanges.

#### Distribution.

China (Hainan Island), Thailand, Malaysia, and Indonesia.

#### Host.

Unknown.

### Rhadinoscelidia lixa

Taxon classificationAnimaliaHymenopteraChrysididae

Hisasue & Mita sp. nov.

7673C925-444D-5BF4-BF5D-6349DBDAC5BA

http://zoobank.org/AF07B6A0-64D8-4C80-A326-0B3F0B163ADC

Figs 1 , 2–5 , 6–7

#### Material examined.

***Holotype*, **♀, Thailand, Phrae Prov. 153 m, Mang Chin Dist., nr. Wiang Kosai NP, 3. V. 2019, R. Ishikawa leg. (Entomological Laboratory, Faculty of Agriculture, Kyushu University).

#### Description of holotype.

**Figure 1.**
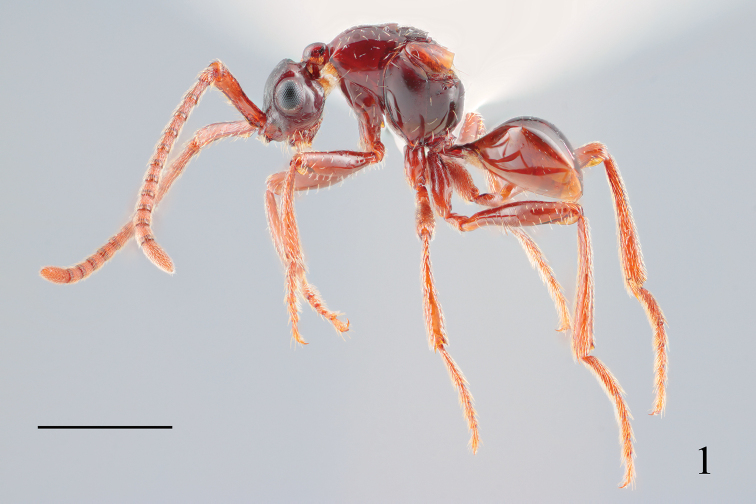
Lateral habitus of *Radinoscelidia lixa* sp. nov. (holotype). Scale bar: 1 mm.

**Female** (Fig. 1). Body 3.0 mm long.

**Figures 2–5.**
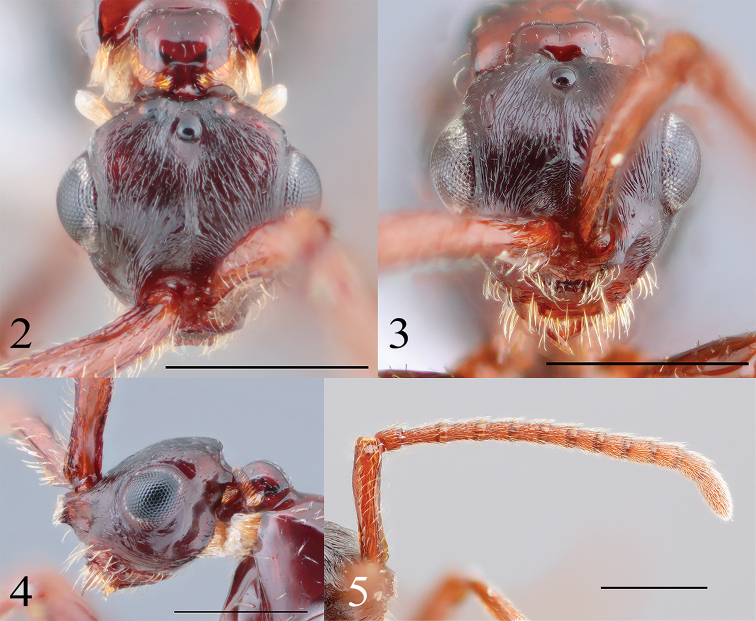
Head of *Radinoscelidia lixa* sp. nov. (holotype) **2** dorsal **3** frontal **4** lateral **5** antenna. Scale bars: 0.5 mm.

***Head*. **Head (Figs 2–4) 1.9 times as long as height in lateral view, 1.3 times as long as maximum width; minimum length between compound eyes 0.7 times as long as head width; frontal projection rectangular in frontal view (Fig. 3); apical margin of frontal projection depressed (Fig. 2); malar space striate; frons striate radially except smooth appressed area in front of midocellus (Fig. 2); low ridge present from around posterior part of inner orbit of eye to posterior depression of vertex; vertex without transverse ridge, deeply depressed posteriorly; cervical expansion curved in lateral view (Fig. 4); temple 3.3 times as long as MOD; POL 2.5 times as LOD; OOL 3.0 times as long as LOD; LOL as long as LOD; scape 4.3 times as long as wide, sparsely punctate, slightly curved, 0.8 times as long as head width (Fig. 5); flange of scape 0.3 times as long as scape length; maximum width of flange 0.6 times as wide as tubular part of scape (Fig. 5); pedicel 1.3 times as long as wide, 0.5 times less than F1; F1–F7 tubular; relative length (width) of F1–F11: 2.4 (1.1): 2.0 (1.2): 1.8 (1.2): 1.6 (1.0): 1.4 (1.0): 1.4 (1.0): 1.4 (1.0): 1.4 (1.1): 1.4 (1.1): 1.4 (1.1): 3.2 (1.2).

**Figures 6, 7.**
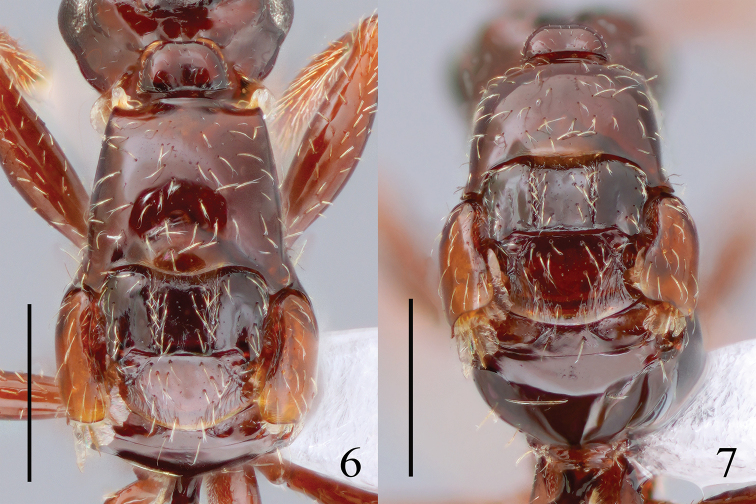
Mesosoma of *Radinoscelidia lixa* sp. nov. (holotype) **6** dorsal **7** lateral. Scale bars: 0.5 mm.

***Mesosoma*. **Mesosoma polished (Figs 1, 6); pronotum 0.9 times as long as maximum width; maximum width of pronotum 1.5 times as wide as posterior width; lateral margin of pronotum without distinct ridge (Fig. 6); mesoscutum 1.1 times as long as wide; tegula polished, 1.5 times as long as wide (Fig. 6); mesoscutum with notauli reaching posterior margin; mesoscutellum polished, 1.6 times as long as maximum width, 4.4 times as long as metanotum length (Fig. 7); length between metanotal depressions 1.5 times as long as length of metanotum (Fig. 7); propodeum smooth; projection weakly developed; dorsal margin of propodeum concave above foramen (Figs 1, 7).

***Legs*. **Legs polished (Fig. 1); femora cylindrical; tibiae slightly flattened, with longitudinal ridge on lower side; forefemur 4.1 times width; foretibia 5.9 times as long as width; midfemur 4.8 times as long as width; midtibia 6.6 times as long as width; hindcoxa 2.4 times as long as hindtrochanter; hindfemur 5.1 times as long as maximum width, 1.5 times as long as head width; hindtibia nearly straight, 8.1 times as long as maximum width (Fig. 1); hindbasitarsus 0.55 times as long as head width; relative length of hindtarsomeres = 3.5: 1.8: 1.7: 1: 2.3; tarsal claws with median tooth.

***Wings*. **Fore and hindwings broken, missing from basal portion (Figs 6, 7).

***Metasoma*. **Metasoma polished and smooth.

***Pilosity*. **Frons with sparse decumbent needle-like setae; eye without setae; frontal projection with dense erect needle-like or cuneate setae (Fig. 2); clypeus with sparse erect needle-like setae (Fig. 3); maxilla with dense decumbent needle-like setae; labrum with dense decumbent needle-like setae; malar space with sparse suberect cuneate or forked setae (Fig. 4); temple with sparse decumbent needle-like setae; vertex behind ocelli with sparse suberect needle-like setae; vertex with ribbon-like setae, shorter than ribbon-like setae on cervical expansion (Figs 2, 4); cervical expansion with sparse decumbent needle-like setae and ribbon-like setae, longer than ribbon-like setae on vertex; upper gena with ribbon-like setae, as long as ribbon-like setae on pronotum (Fig. 4); lower gena with sparse suberect needle-like setae along occipital carina; scape with sparse decumbent needle-like setae and sparse suberect forked setae (Fig. 5); pedicel with dense decumbent needle-like setae; F with dense decumbent needle-like setae, shorter than each F length (Fig. 5).

Anterior margin of pronotum with ribbon-like setae (Fig. 6), as long as those on lower gena; pronotum with sparse suberect cuneate or forked setae in dorsal view (Fig. 6); pronotum with sparse decumbent cuneate or forked setae in lateral view; propleuron with sparse decumbent cuneate or forked setae (Fig. 1); mesoscutum with sparse suberect forked setae (Fig. 6); tegula with sparse suberect forked setae (Fig. 6); mesopleuron with sparse decumbent cuneate or forked setae; metanotum with sparse suberect forked setae (Fig. 7); propodeum with sparse suberect cuneate setae in lateral view (Fig. 7).

Apical half of fore and midcoxae with dense suberect needle-like setae (Fig. 1); femora with sparse erect or suberect cuneate setae (Fig. 1); apical part of coxae with dense decumbent cuneate setae; tibiae with dense decumbent needle-like setae; tarsomeres with dense decumbent needle-like setae.

T2–T3 with sparse decumbent setae (Fig. 1), shorter than setae on S3–S4; S3–S4 with sparse suberect needle-like setae (Fig. 1); T5 with sparse suberect needle-like setae; S5 with dense suberect needle-like setae.

***Coloration*. **Body reddish-brown (Fig. 1); labial palpi, maxillary palpi, and ribbon-like setae brownish yellow; other setae white; flange yellowish brown.

**Male**. Unknown.

#### Etymology.

Named after the Latin ‘lixa’, meaning camp-follower, referring to the wasp walking near the ant’s trail.

#### Distribution.

Thailand (Phrae).

#### Associate.

**Figure 8.**
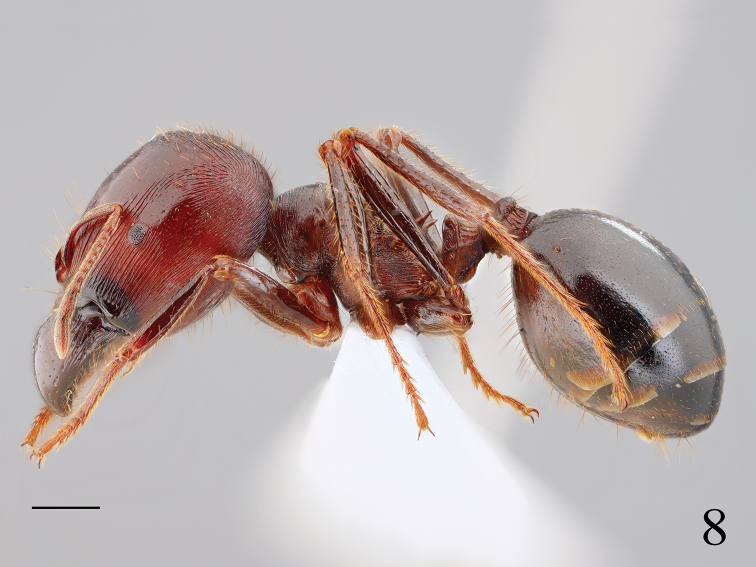
Lateral habitus of *Carebara diversa*. Scale bar: 1 mm.

*Carebara diversa* (Hymenoptera, Formicidae) (Fig. 8).

#### Remarks.

*Rhadinoscelidia lixa* sp. nov. is easily distinguished from other species by the following characters: scape 4.3 times as long as width (over 5 times as long as width in other species); short erect setae of antenna; wide ribbon-like setae on temple wider than those on pronotum (shorter than those on pronotum in other species); shorter ribbon-like setae on cervical expansion (relatively longer in other species); straight hindtibia (slightly or moderately curved hindtibia in other species).

### Key to species of *Rhadinoscelidia* (modified from Kimsey 2018)

**Table:** 

1	Eye small and separated by more than half of its diameter from ocelli in lateral view; hindtarsal claw without tooth; vertex rounded and without transverse carina behind hindocelli	***R. chaesonensis* Kimsey**
–	Eye larger, separated by half its diameter or less from ocelli in lateral view; vertex angulate and with transverse carina behind hindocelli; hindtarsal claw with a median tooth	**2**
2	Scape shorter, 4.3 times as long as wide; pedicel 0.5 times less than F1; OOL 3.0 times as long as LOD; ribbon-like setae of upper gena as wide as those on pronotum; hindtibia almost straight	***R. lixa* sp. nov.**
–	Scape longer, more than 5.0 times as long as maximum width; pedicel as long as or slightly shorter than F1; OOL less than 2.0 times as long as LOD; ribbon-like setae of upper gena much shorter than those on pronotum; hindtibia strongly curved	**3**
3	F1 twice as long as broad; frons with Y-shaped carina extending below midocellus	***R. halimunensis* Ubaidillah**
–	F1 1.3–1.6 times as long as wide; frons with wrinkles or fine carinae diverging from midocellus	**4**
4	Vertex without transverse carina or sharp angle behind ocelli; F11 1.9 times as long as wide	***R. delta* Liu, Yao & Xu**
–	Vertex with transverse carina or sharp angle behind ocelli; F11 1.6–1.7 times as long as wide	***R. malaysiae* Kimsey**

## Discussion

Comparing *Rhadinoscelidia lixa* sp. nov. with the other four species of *Rhadinoscelidia*, the morphological characteristics of *R. lixa* sp. nov. are more conservative, rather similar to those of the genus *Loboscelidia*. In *Loboscelidia*, there is one record from the nest of the ant *Rhytidoponera metallica* (Smith, 1858) (Riek 1970). However, no information on the biology of *Rhadinoscelidia* has been reported until now (Kimsey 2018). According to observations by the collector, the holotype of *R. lixa* sp. nov. stayed at the entrance of the nest of *Carebara diversa*, and it was not attacked by ants. However, the wings of *R. lixa* sp. nov. were cut off from the basal portion (Fig. 7), probably by ants. Even though this is a singular observation, this may explain why *Rhadinoscelidia* has rarely been collected by previous trap-based surveys.

Sometimes, ants attack the wings of the ant-associate wasps. For example, *Paralipsis enervis* (Nees) (Braconidae) and *Bruchopria hexatoma* Kieffer (Diapriidae) have fully developed wings, but wings are eventually cut off by ants after entering the nest (Starý 1966; Loiácono et al. 2002). Similarly, Takada and Hashimoto (1985) reported that *Paralipsis eikoae* (Yasumatsu), an associate of *Lasius japonicus* Santschi (*L. niger* (Linnaeus)), had mutilated wings probably caused by ants. These myrmecophilous wasps show strong adaptation to the host ant, including the nutrition change and the mimicry of the cuticular hydrocarbons. As for *C. diversa*, many ant guests have been reported (Kistner 1983; Kistner and Mcnairn 1991; Geiselhardt et al. 2007). Although there is no evidence of the biological relationship between *R. lixa* sp. nov. and *C. diversa*, this observation could be a foothold for further understanding the little-known biology of *Rhadinoscelidia*.

## Supplementary Material

XML Treatment for Rhadinoscelidia

XML Treatment for Rhadinoscelidia lixa
